# The Dynamics of Nitrous Oxide Emission from the Use of Mineral Fertilizers in Russia

**DOI:** 10.1100/tsw.2001.92

**Published:** 2001-10-12

**Authors:** A.A. Romanovskaya, M.L. Gytarsky, R.T. Karaban, D.E. Konyushkov, I.M. Nazarov

**Affiliations:** Institute of Global Climate & Ecology, Moscow, Russia

**Keywords:** nitrous oxide emission, greenhouse gases, greenhouse gas inventories, synthetic fertilizers, Russia, emission factors

## Abstract

The intensity of nitrous oxide (N2O) emission was considered based on literature data on the single input of mineral N (nitrogen) fertilizers into different agricultural soil types in Russia. Ambient environmental factors exert a combined effect on the process of gaseous nitrogen formation from fertilizers applied. To reduce the uncertainty of estimates as much as possible, only experimental results obtained under conditions similar to natural were selected for the assessments. Mineral nitric fertilizers were applied to soil at a rate of 40 to 75 kg/ha and the N2O emissions were measured for approximately 140 days. Daily average emission values varied from 0.08 to 0.45% of fertilizer nitrogen. Correspondingly, 1.26 and 2.38% of fertilizer nitrogen were emitted as N2O from chernozems and soddy podzols. In 1990, the use of fertilizers in Russian agricultural practices for 53 Gg N2O-N, which equates to approximately 6.1% of global nitrous oxide emissions from nitric fertilizers. Later, the emission dropped because of a decrease in the input of nitric fertilizers to agricultural crops, and in 1998, it constituted just 20.5% of the 1990 level. In the period from 2008 to 2012, the nitrous oxide emission is expected to vary from 0.5 to 65.0 Gg N2O-N due to possible changes in national agricultural development. In the most likely scenario, the use of mineral fertilizers in Russia will account for approximately 34 to 40 Gg N2O-N emissions annually from 2008—2012.

## INTRODUCTION

Nitrous oxide ($N_2O$) is an important greenhouse gas due to its high global-warming potential (310) and large production resulting from a range of human-derived activities. Countries are required to report anthropogenic emissions of greenhouse gases subject to annual inventory submission requirements of the United Nations Framework Convention on Climate Change (UN FCCC)[1]. Synthetic nitric fertilizers are considered the primary source of anthropogenic $N_2O$ emission in agriculture[2]. $N_2O$ is an intermediate product in both nitrification and denitrification processes that are responsible for the gradual oxidation and reduction of nitric compounds in soil. The intensity of soil processes depends on the combined effect of environmental factors such as aeration, pH, content of organic carbon, $NO_3^-$, and $NH_4^+$, etc.[3,4,5,6,7,8]. The majority of environmental factors are dependent on soil properties[6]. Seasonal and daily variation of $N_2O$ emission depends on climatic parameters such as temperature and moisture. The inventory of greenhouse gas emissions is constructed using the Revised 1996 IPCC Guidelines[9] (hereafter referred to as the Guidelines). The Guidelines do not, however, consider $N_2O$ formation in different types of agricultural soils, particularly in regard to the rates of fertilizer application. The latter results in simplification of accounting and high uncertainty values of the estimates obtained. The Guidelines recommend that the countries should develop their own investigations to obtain country-specific $N_2O$ emission factors for specific soils used in agriculture[9].

The aim of this work was to obtain more precise estimates of $N_2O$ emission to the atmosphere from the input of synthetic fertilizers to agricultural soils in Russia. In addition, expected nitrous oxide emission with regard of possible changes in agricultural development was considered.

## OBJECT AND METHODS

The object of the investigation was to estimate the $N_2O$ emissions in Russia for the periods 1990 to 1998 and 2008 to 2012. In their national greenhouse gas inventory, each country should compare the emissions with those obtained for 1990, which is the base year of the Convention[1,9,10]. Thus, emissions were estimated for the periods from 1990 to 1998 and 2008 to 2012 (first commitment period of the Kyoto Protocol)[10]. The anthropogenic $N_2O$ emission from the input of nitric fertilizers was calculated according to equations (1) and (2) taken from[9]:

$$F_{SN} = N_{FERT} \times (1 - Frac_{GASF}) \quad (1)$$

$$E_{N_2O} = F_{SN} \times EF_1 \quad (2)$$

where $F_{SN}$ is part of mineral fertilizer nitrogen applied to agricultural lands that forms the basis for $N_2O$ emission (kg N/year); $N_{FERT}$ is total mineral nitric fertilizer use in active matter (kg N/year); $Frac_{GASF}$ is part of fertilizer nitrogen that emits as $NO_x$ and $NH_3$ (kg $NO_x$-N and $NH_3$-N/kg of total N input); $E_{N2O}$ is anthropogenic $N_2O$ emission from the input of synthetic fertilizer applied (kg $N_2O$-N/year); and $EF_1$ is emission factor for direct $N_2O$ emission from agricultural lands due to fertilization (kg $N_2O$-N/kg N input).

The data on average rates of synthetic fertilization and total mineral nitric fertilizer input to agricultural lands, tree stands, natural hay-lands, and pastures in 1994, 1995, and 1998 were obtained from national statistics[11,12]. The values for 1990, 1993, 1996, and 1997 were estimated from available data on total use of nitric fertilizers and the ratio of their application for crop growing. The values for 1991 and 1992 were obtained through interpolation of data available. The annual input of fertilizers to different soils was calculated based on their part in the general structure of plow lands in Russia and national statistics on the entire use of mineral N fertilizers within the country for 1994, 1995, and 1998[12]. According to the Guidelines, the fraction of synthetic fertilizer nitrogen applied to soil that volatilizes as $NH_3$ and $NO_x$ can be as much as 0.1 $NH_3$-N and $NO_x$-N[9]. This factor was used in the calculation of $N_2O$ emission from agricultural soils in the country.

Because single (spring) input of nitric fertilizers is a common practice in Russia[13], national and international literature data on $N_2O$ emissions from a single application of nitric fertilizers were collected and analyzed to determine the emission factor for different soil types. If the experimental soils corresponded to basic types of agricultural lands of Russia, the experiments were included in the consideration. The experimental results displayed a significant variation, as high as some orders of magnitude[3,5]. To reduce the variability and avoid casual errors, only experiments performed under similar environmental conditions (20 to 25°C, normal atmospheric pressure, and natural pH and field moisture of soil) were analyzed. The rate of applied synthetic fertilizers was another important criterion for initial data selection. According to national statistical reports from 1990 to 1998, the average input of mineral nitric fertilizers in agriculture was no more than 50 kg/ha[11,12]. The input rate exceeded 431 kg/ha several times in 1990, however; for this reason, experiments with nitric fertilizer input less than 500 kg/ha were used in our calculations.

To obtain comparable results, the rates of applied fertilizers were recalculated to kilograms of active matter per hectare, according to Rowell[14]. To perform the recalculation, the depth of plowable layer was assumed to be 20 cm. The values of soil bulk density were taken from a report by the USSR Academy of Sciences[15]. The experimental measurements of $N_2O$ concentration in ground-air layer were recalculated to daily emission of atomic nitrogen ($N_2O$-N) and were stated as the percentage of the total soil and fertilizer nitrogen. Fried and Dean proposed to determine soil nutrient content based on the ratio between the fertilizer input to soil and its uptake by plants[16]. Their formula was applied in case of the lack of data on available nitrogen content in soil. The part of fertilizer nitrogen released as $N_2O$ was expressed as the percentage of total nitrogen applied to soil and assumed as the emission factor for fertilizer nitrogen. The experimental data was treated statistically with the use of standard software. The values of emission factors obtained for different soil types were used in the calculation of $N_2O$ emissions from the input of synthetic fertilizers to agricultural lands in Russia.

In the first commitment period of the Kyoto Protocol, 2008 to 2012, the $N_2O$ emission expected from arable soils was determined based on estimates obtained for the period from 1990 to 1998 and literature data on possible fertilization application rates[17,18]. Three factors were considered for the agricultural sector of Russia: future depression, stabilization at the present state, and sustainable development of agriculture[17]. The nitrous oxide amounts released from nitric fertilizers in 2000, 2001, and 2004 to 2007 were calculated according to the derived emission factors. The values of $N_2O$ emission for 2008 to 2012 were obtained through extrapolation of data available and compared with the 1990 emission level.

## RESULTS AND DISCUSSION

The $N_2O$ emission factors for various agricultural soils were determined based on the data on fertilizer input and mean daily value of $N_2O$ release. The published data of the results of laboratory and vegetating experiments on $N_2O$ emissions from fertilized chernozems and soddy podzols soils were used for the calculations. As mentioned above, the data obtained displayed significant variation, which could be associated with specific conditions of the experiments performed by different investigators. Both external and internal (soil) conditions affect the intensity of $N_2O$ release from soil. Furthermore, the experimental measurements of gaseous nitrogen compounds in the ground-air register an integral result of a complex process that includes interactions between various components of the ambient environment.

Despite the significant variation in the results, analysis of independent data on experimental measurements showed similarity in the dynamics of $N_2O$ daily from different soil types[2,3,4,6,8]. As a rule, the emission increased rapidly soon after the input of fertilizers, and then gradually dropped. A two-year-long experiment by Solov'ev et al.[19] indicated that an abrupt decrease in the nitrous oxide emission occurred on the 40th to 48th days after fertilization. The authors also reported on extremely low $N_2O$ release at the stage of barley maturity[19]. Similar results and trends were obtained from other studies with different crops and on different soils, and were used for calculation of the total period of $N_2O$ emission from soil due to nitrogen fertilizer application[4,20,21]. In more than 95% of experiments, the inputs from 40 to 75 kg/ha and from 160 to 264 kg/ha of mineral nitric fertilizers were considered. These were subdivided into two groups and treated separately. No reliable data exist for fertilizer rates from 75 to 160 kg/ha. It is important to note that the input of 40 to 75 kg/ha of fertilizer nitrogen is currently the typical agricultural practice in Russia, whereas higher rates of nitric fertilizers (160 to 264 kg/ha) are usually applied in Western Europe, the U.S., and Canada[22]. Fig. 1 shows the temporal dynamics of $N_2O$ emissions that correspond to the use of 40–75 (a) and 160–264 kg/ha (b) of synthetic fertilizers. The dots on figure represent results of experimental measurements of integral $N_2O$ emission from both soil and fertilizer nitrogen. Each dot is an average value from several experimental measurements by independent investigators[4,19,20,21]. Despite the significant variation in experimental results, there is a clear trend of increasing in $N_2O$ emission within 40 days after fertilization. The intensity of soil $N_2O$ emission gradually decreased, reaching very low values by the end of the growing season. In general, $N_2O$ emission is regulated by soil microflora activity and the content of available nitrogen in the soil. The uptake of $NH_4^+$ and $NO_3^-$ by crops accounts for principle removal of available soil nitrogen and is associated with the decrease in soil N content during crop growth. The uptake of nitrogen during plant growth occurs rather slowly compared to the rate of soil nitrogen decomposition by microorganisms. Soil microorganisms use $NH_4^+$ and $NO_3^-$ intensively in the month after input (up to 30 to 40 days), and considerable $N_2O$ emissions are associated with microbial activity. The development of crops enhances nitrogen uptake after 30 to 40 days, thereby reducing $N_2O$ release from soil. According to Kudeyarov[7], crops accumulate about 40% of fertilizer nitrogen as ammonium and nitrates during their growth. Decreases in available nitrogen can result when the nitrogen is fixed in soil organic matter (about 25% of N input), leaching of nitrates to ground waters (typically less then 10%), and microbial activity, which removes approximately 15 to 25% of fertilizer nitrogen[7,23]. These ratios are applicable for both soluble soil and fertilizer nitrogen[24]. These quotients are described by the following formula:

$$y(t) = k_o e^{-t/\tau 1}(1 - e^{-t/\tau 2}) \quad (3)$$

where y(t) is $N_2O$ daily emission, the percentage of nitrogen soil content after fertilization $k_o$ is the coefficient that regulates $N_2O$ emission at large values of t ($t \rightarrow 8$); $\tau_2$ is the mean time period necessary for reaching K(t) of magnitude 0.63 $k_o$, days; and $\tau_1$ is an average period of nitrogen transformation in the upper soil layer, days.

Anthropogenic impact disturbed the natural nitrogen balance in soil and increased $N_2O$ release[8]. The input of synthetic fer-

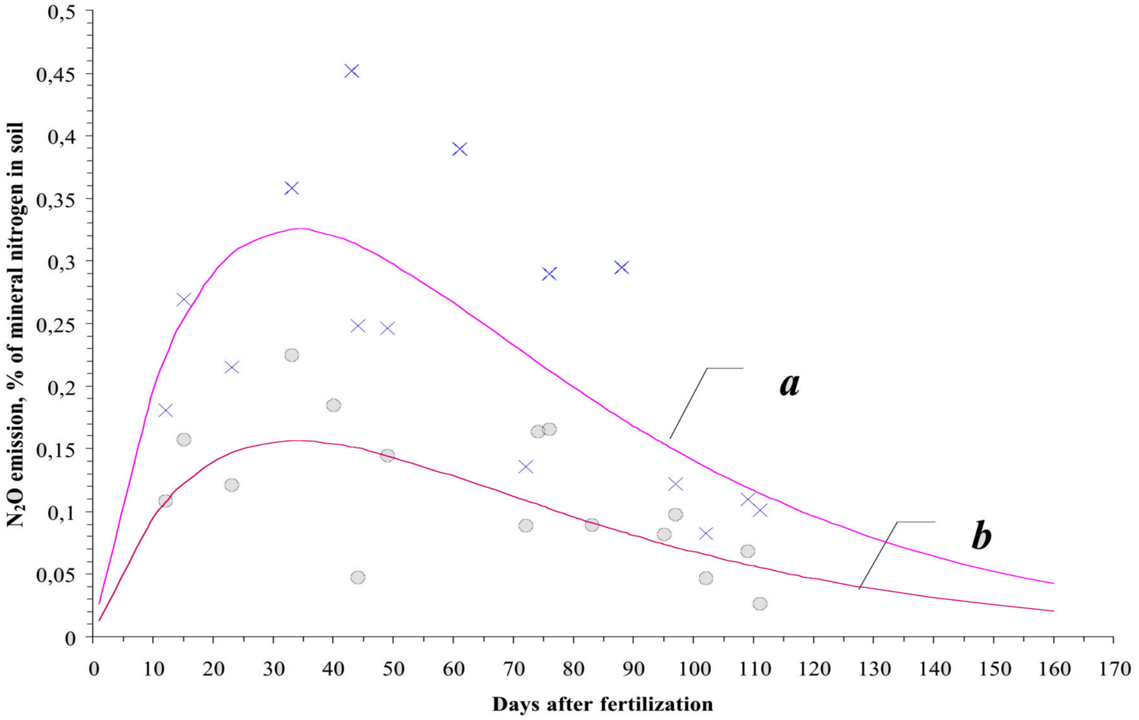

**FIGURE 1.** The dynamic of $N_2O$ emission from a single input of synthetic nitric fertilizers derived according to generalized literature data.[7, 20, 21, 25] a, input from 40 to 75 kg/ha; x, mean daily $N_2O$ emission from the input of 40 to 75 kg/ha (experimental literature data); b, input from 160 to 265 kg/ha; o, mean daily $N_2O$ emission from the input of 40 to 75 kg/ha (experimental literature data).

tilizers is usually followed by a high mineralization of soil organic matter and raises the content of available soil nitrogen[24]. Soils become a source of human-induced $N_2O$ emission that includes both the emission of fertilizer nitrogen and mineralized soil nitrogen. The function (3) is a simple simulation model of $N_2O$ release from soils after the input of mineral nitric fertilizers. It describes regularities in nitrous oxide formation in human-disturbed soils and gives their quantitative estimates.

Based on regression analysis of experimental data on Fig. 1 the following equations were derived:

$$y_1(t) = 1{,}6e^{-t/45}(1 - e^{-t/60}), \quad \text{where } r = 0{,}723 \quad \text{for the input 40 to 75 kg/ha} \tag{4}$$

$$y_2(t) = 0{,}77e^{-t/45}(1 - e^{-t/60}), \quad \text{where } r = 0{,}623 \quad \text{for the input 160 to 264 kg/ha} \tag{5}$$

Integration by y(t), $(t \rightarrow 8)$ provides nitrous oxide emission for the period and part of its total release after a single application of nitrogen-containing fertilizers. The input of 40 to 75 kg/ha of fertilizer nitrogen value ($Y_1$) contributes to 30% of soil nitrogen lost as $N_2O$, whereas 160 to 264 kg/ha of fertilizer nitrogen ($Y_2$) results in 15% of nitrogen losses. According to Smirnov[23] and Kudeyarov[7], gaseous loss of soil and fertilizer nitrogen ranges from 15 to 25%. Hence, our results are in strong agreement with their data. As shown by the values $Y_1$ and $Y_2$ and curves (a) and (b) on Fig. 1, the input of higher rates of synthetic fertilizers depresses relative nitrous oxide emission. It is possible to assume that the increase in rates of added fertilizers intensifies microbial activity up to a certain limit. After that, the population growth of microorganisms becomes restricted by the effect of external factors such as availability of oxygen and nutrition substrate. In that case, the growth of soil nitrogen content corresponds to the decrease in relative $N_2O$ emission. Evidently, extremely high soil nitrogen content can inhibit assimilation of nutrients by microorganisms. Thus, it is possible to expect a further decrease in relative $N_2O$ emission from the input of nitric fertilizers at rates higher than 264 kg/ha.

Based on the exponential reduction of soil nitrogen content, the efficient period of nitrous oxide emission ($t_{ef}$) was determined. It was assumed as 90% of the maximum $N_2O$ release after fertilizer input. The emission rates reached low activity values after approximately the 140th day from the input of 40–75 and 160–264 kg/ha of fertilizer nitrogen, which is in agreement with the data by Muller et al.[25]. The rates of nitric fertilizers from 40 to 75 kg/ha are used in Russia. Assumptions can be made that in the Russian Federation, $N_2O$ emission from agricultural lands lasts almost for 140 days. This value was used in the calculation of emission factors for different soil types. Based on total duration of emission and its daily mean values for each soil, the total loss of nitrous oxide from nitric fertilizers were calculated and expressed as percentages. This estimate gives the emission factor $EF_1$. To obtain mean daily emissions, the values of $N_2O$ released from fertilizer nitrogen obtained for similar ambient conditions were taken and expressed as percentage of nitric fertilizer input to make them fully comparable and suitable for subsequent calculations of mean daily emissions. The results of the calculations are given in Table 1.

The multiplication of the $N_2O$ daily emission value by the efficient period of its release gives the total $N_2O$ emission from synthetic nitric fertilizers (Table 1). The expressed (in decimals) estimate is the emission factor ($EF_1$) for chernozems and soddy-podzolic soils, respectively, 0.0126 and 0.0238 kg $N_2O$-N/kg of fertilizer N input. The numbers show the decimals (rate) of fertilizer nitrogen that emits $NO_2$ from the soil. The difference can be explained by soil properties that affect the nature of the fertilizer transformation. In chernozems, intensive transformation results in rapid decomposition of nitrogen and its subsequent assimilation by plants and microorganisms. Intensive microbial activity promotes full reduction of $NO_3^-$ to $N_2$ and lowers $N_2O/N_2$ ratio in denitrification[8]. Decreases in denitrification intensity in acidic soddy-podzolic soils causes the intermediate $N_2O$ formation to increase[6]. In addition, low pH, high moisture, and poor aeration in soddy-podzolic soils can intensify chemo-denitrification, which also produces $N_2O$. Therefore, the emission of $N_2O$ is lower in chernozems than in soddy- from the soil.podzolic soils. The IPCC recommends a default emission factor of 0.0125 kg $N_2O$-N/kg N[9]. The value derived for chernozems is close to the recommended default, whereas the emission factor for soddy-podzolic soils is almost two times higher.

Based on the $EF_1$ values obtained and data on the input of synthetic fertilizers, $N_2O$ emission from chernozems and soddy-podzolic soils was calculated with the use of equations (1) and (2). At present, the lack of data on other soils makes it impossible to derive $N_2O$ emission factors for them; therefore, emissions were estimated with the use of the IPCC default value as proposed in the Guidelines[9]. Table 2 presents $N_2O$ emission from various soil types and total emission of nitrous oxide caused by the input of synthetic fertilizers in Russia. In 1990, the global $N_2O$ emission from the use of nitric fertilizers was 870 Gg $N_2O$-N[9,26]. The input of fertilizer nitrogen to agricultural lands of Russia in 1990 caused the release of almost 53 Gg $N_2O$-N, which is 6.1% of the total global emission from nitric fertilizers (Table 2). Contribution of chernozems to $N_2O$ emission was more than 50%. Later, the emission significantly reduced, and in 1997 it accounted for no more than 0.4% of the

**TABLE 1**
**Mean Daily Emissions and Total Loss of $N_2O$ from Nitric Fertilizers Applied to Agricultural Lands, $N_2O$-N, Percentage of Fertilizer N**

| Soil Type | Mean Daily $N_2O$ Emission | Total $N_2O$ Emission |
|---|---|---|
| Chernozem | 0.009 | 1.26 |
| Soddy podzol | 0.017 | 2.38 |

global value. In 1998, human-induced $N_2O$ emission by agricultural lands in Russia comprised about 20.5% of the 1990 level. The decrease between 1990 and 1998 is associated with reduced input of synthetic fertilizers in national agriculture. From 1995 to 1998, there was a relative stabilization of nitrous oxide emission in Russia (Table 2).

According to the Kyoto Protocol of the Convention[10], the Russian Federation faces two tasks: 1) exerting more control over its greenhouse gas emissions in various sectors of the economy, including agriculture; and 2) not exceeding the 1990 emission level. Figs. 2 and 3 present possible $N_2O$ emission trends based on different scenarios of agricultural sector development. As shown in Fig. 2, in the event of a future depression in the agricultural sector of the country during 2008 to 2012, the $N_2O$ emission could to between 1 and 0,5 Gg $N_2O$-N, respectively. Reduction of arable lands, decreased rates of applied synthetic fertilizers, and the resulting decrease in agriculture production could be the major reasons for reduced $N_2O$ emission. Another change in national agricultural development is stabilization at the present state of crop production. In the period from 2008 to 2012, the nitrous oxide emission will be saved on 1997 to 1998 level, or about 11 to 13 Gg $N_2O$-N. Hence, $N_2O$ emissions from the input of synthetic fertilizers will not exceed 1990 level.

Sustainable agricultural development would mean growth of crop productivity with increasing fertilizer use. High input of nitric fertilizers will be associated with increases in the nitrous oxide emissions that will rapidly reach the 1990 level. However, the time period necessary to reach the 1990 fertilizer application levels is not determined precisely in the current economic literature[17,18]. Thus, two possibilities (intensive and gradual development) can be considered. Results of expected nitrous oxide emissions are presented in Fig. 3. In the event of major change, the input of fertilizer nitrogen to agricultural lands of Russia in the period from 2008 to 2012 will cause the release of 52 and 65 Gg $N_2O$-N, respectively, or approximately 98 and 112% of the 1990 level. However, gradual development is the most reliable scenario for $N_2O$ emission from the use of mineral fertilizers in Russia. It may contribute 34 and 40 Gg $N_2O$-N in 2008 and 2012, respectively, or 64 and 75% of the 1990 level. In that event, nitrous oxide emissions from the input of synthetic fertilizers will not exceed the 1990 level.

**TABLE 2**
**The $N_2O$ Emission from Agricultural Lands of Russia (Gg $N_2O$-N/year)**

| Year | Chernozems | Soddy-Podzolic Soils | Other Soils | Total |
|---|---|---|---|---|
| 1990 | 30.03 | 13.01 | 9.85 | 52.89 |
| 1991 | 26.09 | 11.3 | 8.56 | 45.95 |
| 1992 | 21.62 | 9.36 | 7.09 | 38.07 |
| 1993 | 15.7 | 6.8 | 5.15 | 27.65 |
| 1994 | 9.11 | 3.95 | 2.99 | 16.05 |
| 1995 | 6.98 | 3.02 | 2.29 | 12.29 |
| 1996 | 6.87 | 2.98 | 2.26 | 12.11 |
| 1997 | 7.15 | 3.1 | 2.35 | 12.6 |
| 1998 | 6.17 | 2.67 | 2.02 | 10.86 |

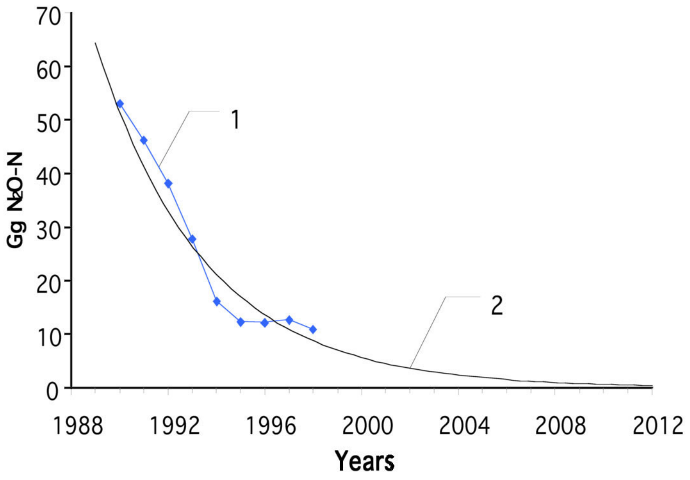

**FIGURE 2.** Possible $N_2O$ emission from agricultural lands of Russia in case of future depression. 1, nitrous oxide emission for period 1990 to 1998; 2, trend line.

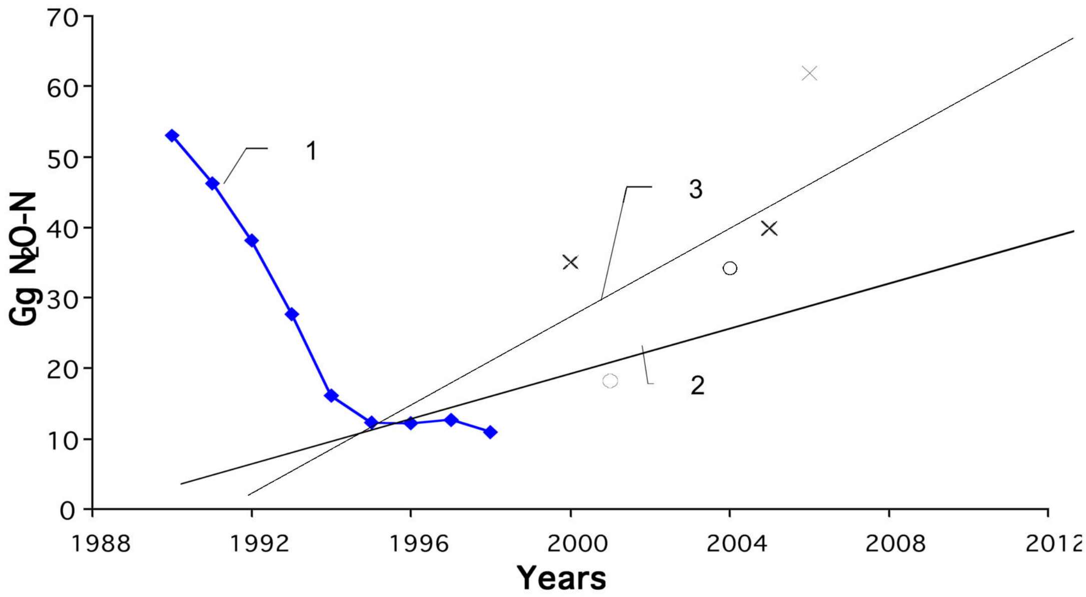

**FIGURE 3.** Possible $N_2O$ emission from agricultural lands of Russia in case of sustainable development. 1, nitrous oxide emission for period 1990 to 1998; o, data for trend line 2 (gradual development); x, data for trend line 3 (intensive development).

Based on the sustainable land use, $N_2O$ emission can be limited by choice of fertilization rate, periods, and methods of fertilizer input. Meanwhile, gaseous loss of nitrogen reduces efficiency of fertilizer application. Our results show that increased input of nitric fertilizers may decrease the rate of gaseous nitrogen losses as $N_2O$. Therefore, the choice of optimum fertilization rate for most economical usage of nitrogen is possible. An increase in the rates of synthetic fertilizer application in Russia may contribute to the sustainable development of the national agricultural sector.

## CONCLUSIONS

The input of 40 to 75 kg/ha contributes up to 30% of $N_2O$ emission of available soil and fertilizer nitrogen within the vegetation period. The input of higher rates of synthetic fertilizers (160 to 264 kg/ha) caused lower emission (15%). Evidently, the higher nitrate concentrations intensify microbiological activity up to certain limits and, therefore, decrease the relative $N_2O$ emission from soil. The majority of nitrous oxide is released within 140 days after fertilizer application in both the levels of fertilizer application studied (40–75 and 160–264 kg/ha of fertilizer nitrogen).

The obtained values of $N_2O$ emission factor for fertilizer nitrogen applied to chernozems and soddy-podzolic soils are 0.0126 and 0.0238 kg $N_2O$-N/kg N, respectively. The values depend on soil properties that specify the process of fertilizer transformation. Low values of pH, high moisture, and poor aeration in soddy-podzolic soils can stimulate $N_2O$ production via biochemical and chemical processes.

In 1990, human-induced emission of $N_2O$ from the use of mineral fertilizers in Russian agriculture was 53 Gg $N_2O$-N. It comprised more than 6.1% of global $N_2O$ emission from this category source. In later years, the national emission level decreased, and in 1997 it accounted for approximately 0.4% of the global level. In 1998, the anthropogenic $N_2O$ emission by agricultural lands in Russia was 21% of the 1990 level, as a result of considerable reduction (more than 70%) in the input of synthetic fertilizers from 1990 to 1998.

In the period from 2008 to 2012, the nitrous oxide emission is expected to vary from 0.5 to 65.0 Gg $N_2O$-N with regard to possible changes in national agricultural development. Three changes were considered: future depression, stabilization at the present level, and sustainable development (intensive and gradual) of the agriculture. The most reliable scenario for $N_2O$ emission from the use of mineral fertilizers in Russia is gradual development. It may contribute to 34 and 40 Gg $N_2O$-N in 2008 and 2012, respectively, which comprises 64 and 75%, respectively, of the 1990 level. In that event, nitrous oxide emission from the input of synthetic fertilizers will not exceed the 1990 level.

## ACKNOWLEDGEMENTS

The authors acknowledge financial support provided by the Russian Foundation for Basic Research, project no. 99-05-64130.

## References

1. UN-FCCC. (1998) UN Framework Convention on Climate Change. UNEP/IUC, 30.
2. Bouwman, A.F. (1990) *Exchange of Greenhouse Gases between Terrestrial Ecosystems and the Atmosphere, Soils and*

*Greenhouse Effect*. Bouwman, A.F., Ed. Wiley, New York. pp. 61–127.

3. Borisova, N.I. and Zersalov, V.V. (1966) The fertilizer nitrogen loss in way $N_2$ and $N_2O$. *Agrokhimiya*. **1**, 13–19. (In Russian)
4. Christensen, S. (1985) $N_2O$-formation during soil cropping. *Denitrification in the Nitrogen Cycle*. Plenum, New York. pp. 135–144.
5. Gantimurova, N.I. (1984) The intensity of microbial transformations of nitrogen compounds in soils of a steppe ecosystem and agrosystem. In *The Agrocenoses of Steppe Zone*. Nauka, Novosibirsk. pp. 81–93. (In Russian).
6. Granli, T. and Bockman, O.C. (1994) Nitrous oxide from agriculture. *Norwegian J. Agric. Sci*. **12**, 7–125.
7. Kudeyarov, V.N. (1989) *The Nitrogen Cycle in Soil and Fertilizer Efficiency*. Nauka, Moscow. 216. (In Russian)
8. Stepanov, A.L. (2000) Microbial Transformation of Nitrous Oxide in Soil, Abstract of Doc. Sci. (Biol.) Dissertation, Moscow, 49. (In Russian)
9. Revised 1996 IPCC Guidelines for National Greenhouse Gas Inventories. (1997) IPCC–OECD–IEA, Paris.
10. Kyoto Protocol to the Convention on Climate Change. (1998) UN-FCCCC, UNEP/IUC, 33.
11. Anonymous (1998) *Agriculture in Russia. A Statistical Textbook*. Goskomstat, Moscow. 448 pp. (In Russian)
12. Anonymous (1999) *Application of Mineral and Organic Fertilizers in 1998.* Goskomstat, Moscow. 81 pp. (In Russian).
13. Anonymous (1965) *The Technology Process Charts for Crop Cultivation*. Lenizdat, Leningrad. 228 pp. (In Russian)
14. Rowell, D.L. (1998) *Soil Science: Methods and Applications.* Longman. 1994. Translated under the title: Pochvovedenie, metody i ispol'zovanie, Moscow: Kolos, 486. (In Russian)
15. *The Agrochemical Characteristics of USSR Soils, Vols. 1–15*. (1962–1976) USSR Academy of Sciences, Moscow. (In Russian).
16. Fried, M. and Dean, L. (1952) A concept concerning the measurement of available soil nutrients. *Soil Sci.* **73(4)**, 263–271.
17. Bespakhotny, G. (1999) The dodges of stabilization and development in agrarian complex. *Agriculture Economic of Russia*, **11**, 5. (In Russian)
18. Gorbachev, A.L., Chernenko, U.D., Klassen, P.V., and Brodski, A.A. (1998) Production outlooks of broad assortment of mineral fertilizers. *Agrokhim. Vestnik* **4**, 15–17. (In Russian)
19. Solov'ev, G.A., et al. (1988) Optimization of the water budget of soddy-podzolic soil at the application of different forms and rates of nitrogen fertilizers. In *Optimization of Soil Water and Nitrogen Regimes*. Lomonosov State University, Moscow. 139–149. (In Russian)
20. Stefanson, R.C. and Greenland, D.J. (1970) Measurement of nitrogen and nitrous oxide evolution from soil-plant systems using sealed growth chambers. *Soil Sci*. **109(3)**, 203–206.
21. Umarov, M.M., et al. (1996) Nitrogen-fixing and denitrifying activity of Gray Forest soil and nitrogen transformation upon nitrogen fertilization, *Agrokhimiya* **2**, 3–10. (In Russian)
22. Matthews, E. (1994) Nitrogenous fertilizers: global distribution of consumption and associated emissions of nitrous oxide and ammonia. *Global Biochem. Cycles*. **8(4)**, 411–439.
23. Smirnov, P.M. (1982) *Problems of Nitrogen Agrochemistry*. Timiryazev Agricultural Academy, Moscow. 74. (In Russian)
24. Bashkin, V.N. (1987) *Nitrogen Agrogeochemistry*, NCBI AN USSR, Puschino. 270 pp. (In Russian)
25. Muller, C., Sherlock, R.R., and Williams, P.H. (1997) Mechanistic model for nitrous oxide emission via nitrification and denitrification. *Boil. Fertil. Soils*, **24(2)**, 231–238.
26. Mosier, A.R. and Kroeze, C. (1998) A new approach to estimate emissions of nitrous oxide from agriculture and its implications to the global $N_2O$ budget. *Newsl. Int. Global Atmos. Chem. Proj.* **12**, 17–25.

| | | |
|---|---|---|
| Received: | July | 10, 2001 |
| Revised: | September | 3, 2001 |
| Accepted: | September | 3, 2001 |
| Published: | October | 12, 2001 |
